# Visual Stimuli Evoked Action Potentials Trigger Rapidly Propagating Dendritic Calcium Transients in the Frog Optic Tectum Layer 6 Neurons

**DOI:** 10.1371/journal.pone.0139472

**Published:** 2015-09-28

**Authors:** Gytis Svirskis, Gytis Baranauskas, Natasa Svirskiene, Tatiana Tkatch

**Affiliations:** Neurophysiology laboratory, Neuroscience Institute, Lithuanian University of Health Sciences, Kaunas, Lithuania; Institut Curie, FRANCE

## Abstract

The superior colliculus in mammals or the optic tectum in amphibians is a major visual information processing center responsible for generation of orientating responses such as saccades in monkeys or prey catching avoidance behavior in frogs. The conserved structure function of the superior colliculus the optic tectum across distant species such as frogs, birds monkeys permits to draw rather general conclusions after studying a single species. We chose the frog optic tectum because we are able to perform whole-cell voltage-clamp recordings fluorescence imaging of tectal neurons while they respond to a visual stimulus. In the optic tectum of amphibians most visual information is processed by pear-shaped neurons possessing long dendritic branches, which receive the majority of synapses originating from the retinal ganglion cells. Since the first step of the retinal input integration is performed on these dendrites, it is important to know whether this integration is enhanced by active dendritic properties. We demonstrate that rapid calcium transients coinciding with the visual stimulus evoked action potentials in the somatic recordings can be readily detected up to the fine branches of these dendrites. These transients were blocked by calcium channel blockers nifedipine CdCl_2_ indicating that calcium entered dendrites via voltage-activated L-type calcium channels. The high speed of calcium transient propagation, >300 μm in <10 ms, is consistent with the notion that action potentials, actively propagating along dendrites, open voltage-gated L-type calcium channels causing rapid calcium concentration transients in the dendrites. We conclude that such activation by somatic action potentials of the dendritic voltage gated calcium channels in the close vicinity to the synapses formed by axons of the retinal ganglion cells may facilitate visual information processing in the principal neurons of the frog optic tectum.

## Introduction

In lower vertebrates the optic tectum processes most visual information received by retina [1–4]. In frogs most motor behaviors are initiated in the optic tectum, including prey catching danger avoidance [1]. Although in mammals, especially in primates humans, visual cortex hles most visual information [5], many visually triggered behaviors such as covert overt attention shifts the maintenance of visual stability during saccades is performed by the superior colliculus, a homolog of the optic tectum [6–10]. The structure the function of the superior colliculus the optic tectum is conserved across such distant species as frogs, birds primates [4, 11–13]. Both in primate superior colliculus in the optic tectum of frogs neurons of the superficial layers receive direct retinal input from the retinal ganglion cells while deeper layer neurons send axons to the motor centers [2, 11, 14–16]. Because of these similarities, it is possible to improve our knowledge about the function of the superior colliculus in mammals by studying optic tectum in lower vertebrates birds that permit application of techniques, which may be difficult to use in the mammal superior colliculus [17–19].

In amphibians most of visual information is processed by principal, pear-shaped neurons of layers 6 8 that possess elaborate dendritic trees [2, 3, 15, 16]. Retinal ganglion cell axons make synapses on these dendrites [20, 21], thus detailed knowledge about the properties of these dendrites is important for understing visual information processing in amphibians;, by homology, in the superior colliculus of mammals.

Active dendrites with voltage-dependent ion channels can perform elaborate processing of synaptic inputs, thus, increase information processing capacity of neurons [22–24]. Past work has hinted that dendrites of tectal pear shaped neurons may support active action potential propagation. For instances, the axons of the tectal neurons originate, as a rule, from the dendritic branches of these neurons [2, 15]. Nevertheless, in somatic recordings most action potentials have large amplitude [25, 26], suggesting that they propagate from their putative dendritic site of origin actively, without a significant attenuation. In addition, in the upper layers of the optic tectum, devoid of somatic bodies of the neurons, extracellular recordings suggested the presence of action potentials calcium dependent dendritic depolarizations [27, 28]. Although dendritic calcium transients caused by both NMDA receptor containing synapses action potentials have been recorded during visual stimulation in the developing frogs [17], to the best of our knowledge, no data has been published on such signals in adult frog neurons. We performed calcium ion imaging in our adult frog eye-tectum preparation that permits patch clamp recordings while visual stimulus is presented. Our preliminary results showed that visual stimuli could readily induce large calcium concentration transients in the dendritic branches of the layer 6 neurons. Since the vast majority of these transients were associated with action potentials, we investigated in more detail the nature of these transients. The obtained results are consistent with a hypothesis that calcium enters dendritic branches during active action potential propagation due to the opening of L-type voltage-gated calcium channels.

## Materials and Methods

For experiments we employed an integrated frog eye-tectum preparation [29]. All procedures were carried out in accordance with the European Communities Council Directive of 24 November 1986 regarding the protection of animals used for experimental other scientific purposes (86/609/EEC) were approved by the Animal Care Use Committee of the State Food Veterinary Service of Lithuania (No. 0167 of 31 October 2007). Briefly, a frog (*Rana temporaria*) was anesthetized by immersion into 0.1% tricaine (MS-222) solution [30] while anesthesia levels were checked by a prod (pinch). Then a transcardially perfused frog was decapitated the head with intact eyes placed in an ice-cold oxygenated high magnesium (10 mM) Ringer solution. To access the brain, the roof of the skull was removed the pia mater overlaying the tectum was retracted. Next the preparation was glued with cyanoacrylate on its ventral cranial surface placed in a vibroslice chamber. The caudal part of the tectum was cut off in order to expose deeper tectal layers 6 8 containing neuronal cell bodies. The frontal part of the telencephalon was also removed for ethical reasons for a better access of Ringer solution to the brain ventricles. The recording chamber was filled with 25 ml of normal Ringer solution consisting of (in mM) 113 NaCl, 3 KCl, 20 NaHCO_3_, 1 MgCl_2_, 1.3 CaCl_2_, 5.5 glucose, pH 7.4, which was continuously supplied at a flow rate of 15 ml/min. The left eye was oriented toward the wall of the test chamber, made of a glass plate with a computer monitor sting behind it. Whole cell recordings were made with patch pipettes filled with an internal solution consisting of (mM): 130 K-gluconate, 10 KCl, 10 HEPES, 1 ATP (pH = 7.3). In the bath, pipette resistance was 20–40 MΩ; layer 6 neurons were patched under visual guidance achieved with an oblique illumination [31]. We employed the oblique illumination because the frog head is not transparent for stard IR-DIC optics.

**Fig 1:**
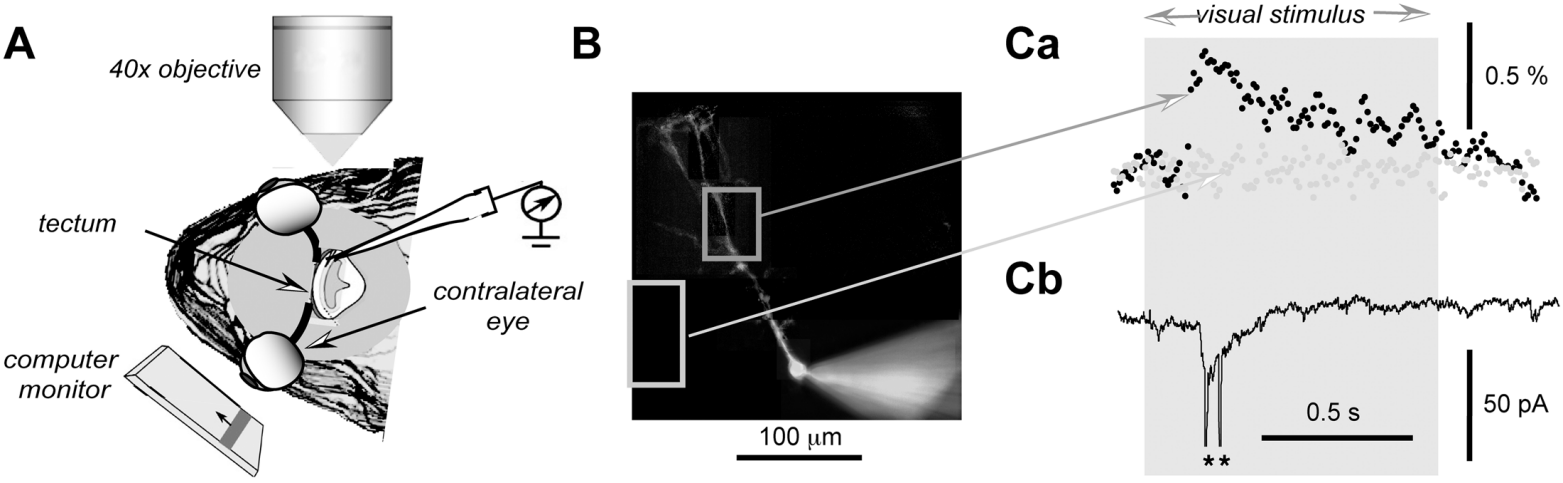
Experimental set-up for recording of dendritic calcium concentration changes during visual stimulation. ***A***. A schematic representation of the frog head placed under 40x long working distance objective for fluorescence imaging of patch-clamped neurons in the frog optic tectum. The visual stimulus was presented on the computer monitor located on the other side than the recorded neuron. ***B***. A stack of 15 images at different focus depth was used to reconstruct this picture of a typical layer 6 neuron recorded in the frog optic tectum. Light grey squares represent areas used for calculation of the time-course of the fluorescence intensity shown in ***Ca***. ***Ca*** represents two fluorescence intensity traces obtained during visual stimulus, a 1 s long dimming of the monitor, indicated by light grey background. Black filled circles represent the proximal dendritic branches of a neuron shown in B light grey filled circles represent a background trace obtained from the area with no dendritic branches of the recorded neuron. Here, to facilitate the comparison with the background trace, both the proximal dendrite the background fluorescence signals were normalized to the overall baseline fluorescence, background plus signal. Meanwhile in Figs 3 and 4 the fluorescence signal was normalized only to the image signal as shown in Fig 2. ***Cb*** represents currents recorded from the same neuron during visual stimulation. The asterisks mark currents generated by action potentials.

Two types of visual stimuli were used. First, dimming of the whole computer screen or an OFF stimulus [3]. Second, a ~20° wide black circle was presented in the center of the receptive field [29]. All images were presented on a computer monitor (frame rate 75 Hz, 35 cm by 17 cm) located 10 cm from the left eye of the frog, with the recording chamber kept relatively dark (Fig 1A) resulting in a very wide visual field (the full screen subtended >120° at the eye). At the center of the screen 1 cm corresponded to ~5.7° of visual angle. The monitor had 1024 X 768 image pixels or >30 pixels/cm corresponding to a minimal stimulus size of <0.3°, below the reported retinal sensitivity of ~1.5° in frogs [32]. Images were generated by employing an open source software package PsychoPy [33] controlled by a program written in the Labview environment (National Instruments, Austin, TX, USA).

Imaging was performed with an or Neo high-resolution (5.5 Mpixel) high-speed (up to >1000 frames per second) sCMOS video camera (or Technology Ltd., Belfast, UK). Calcium sensitive dye OGB-1 (Molecular Probes—Life Technologies-Thermo Fisher Scientific, Waltham, MA, USA), was loaded via patch pipette at 1–2 mM concentration. In some case, to aid the localization of distal dendrites Alexa Fluor 488 hydrazide, 0.4 mM (sodium salt, Life Technologies-Thermo Fisher Scientific, Waltham, MA, USA) was added to the pipette solution. A comparison of fluorescence responses obtained with pipettes filled with OGB-1 alone with OGB-1 plus Alexa Fluor 488 showed no large differences in the calcium response relative amplitude in the proximal dendrites suggesting that only a small fraction of fluorescence came from Alexa Fluor 488. Thus data from both types of pipette filling were pooled together for analysis. At least 20 min after the seal rupture was allowed to perfuse the cell with the dye solution. Fluorescence images were obtained by illuminating with a high power 480 nm LED light source (Prizmatix Ltd., Givat-Schmuel, Israel) via U-MWIBA3 b pass 510–550 nm filter (Olympus Corporation, Tokyo, Japan). Care was taken to minimize eye exposure to the LED light because preliminary tests showed that LED light alone due to scattering can evoke visual responses in tectal neurons or even make eye non responsive, presumably due to retina damage. Therefore a shield of non-transparent material was placed on the top of the frog head with a hole for tectum only. In addition, all chamber walls the holder of the head were black painted to minimize reflections. Finally, the LED light was limited to the recording site by a diaphragm.

Images were acquired with Solis software (or Technology Ltd., Belfast, UK) stored on the disk for further analysis. Synchronization with patch clamp recordings was achieved by triggering camera from the same voltage generator that was used for voltage clamp experiments. In addition, we verified synchronization by aligning an artifact in the fluorescence traces caused by screen dimming. These artifacts were eliminated from the fluorescence traces during subtraction of the background fluorescence signal. Image analysis was performed with Solis analysis software Igor Pro 6 program (Wavemetrics Inc, Lake Oswego, OR, USA) by employing image analysis package custom written subroutines. The time course of the fluorescence signals was corrected for bleaching background fluorescence by subtracting of the fluorescence signals from areas without dendritic branches by subtracting a single exponential function obtained during fit of the baseline obtained before visual or electrical stimulation of the neuron.

All statistical data are presented as average + SEM, non-parametric Kruskal-Wallis tests were used to obtain p values unless noted otherwise.

## Results

For visualization of the dendritic calcium concentration changes, 22 optic tectum neurons from 15 *Rana Temporaria* frogs were filled with a calcium sensitive dye OGB-1 [34]. Fluorescence images of the recorded cells could be readily obtained via an objective located vertically on the top of the optic tectum (Fig 1B) while the computer monitor was located on the other side than the recording pipette in front of the contralateral eye, the axons from which enter mainly contra-laterally to the optic tectum [2].

In all cells tested, OGB-1 fluorescence transients, recorded following the presentation of a visual stimulus on the computer monitor, could be detected only in the areas with the images of the dendritic branches of the recorded neuron while no changes in the fluorescence could be observed outside the dendritic field of the recorded neuron (Fig 1C). For most of our recordings we employed voltage clamp even though we did not have a complete voltage control in the dendrites as can be seen from action potential generated currents (Fig 1C, asterisks). We employed voltage clamp because it permits a better separation of the somatic dendritic signal components the dendritic membrane charging is faster in voltage clamp mode compared to current clamp [35]. Thus, we were able to determine the timing of action potential currents with more confidence.

**Fig 2:**
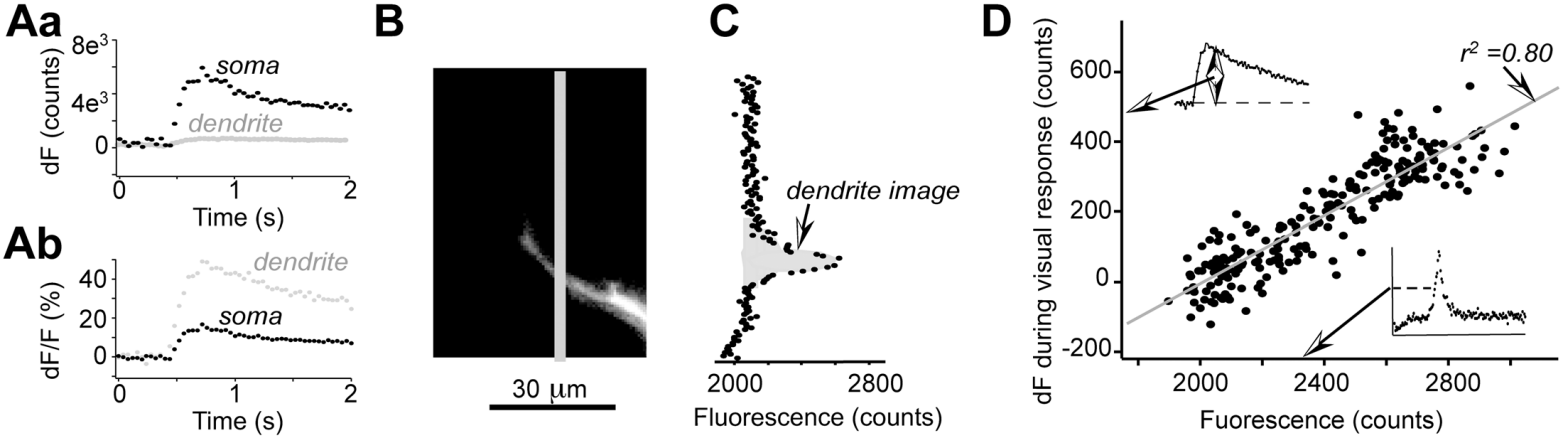
The recorded calcium transients could be detected only on the dendritic branches of the patch-clamped neuron. ***A***. Calcium indicator OGB-1 fluorescence signals in response to several action potentials are shown for soma (black dots) a proximal dendritic branch (grey dots). In Aa absolute values of intensity are shown while in ***Ab*** fluorescence intensity values were normalized to the soma the dendritic branch image intensity values. ***B***. A section of the proximal dendritic branch is shown as an image its fluorescence intensity profile along grey line is shown in ***C***. In ***D*** for each pixel the image fluorescence intensity value before stimulus is plotted against a change in the fluorescence intensity during stimulation. A grey line represents a linear fit of all data points.

To further verify that the obtained signals were originating from the dendritic branches of the recorded neurons, we investigated how the amplitude of the fluorescence signal transient correlates with the fluorescence image amplitude for each image pixel (Fig 2). In absolute fluorescence counts, calcium transient amplitudes were much larger in the soma than in the dendrites (Fig 2A). On average, for the same illumination conditions, the amplitude of the OGB-1 fluorescence change induced by an ‘OFF’ stimulus was 1075 ± 726 counts in the soma 163 ± 67 in the proximal dendrites, 50–100 μm from the soma (n = 6, p <0.05). However, if we normalize these changes to the average image amplitude (shown as a grey area in Fig 2C), the relative fluorescence change was much larger in the dendrites, 40 ± 9%, than in the soma, 6.1 ± 2.2%, (n = 6, p < 0.005). Since we did not use confocal microscope, a fraction of such a strong somatic signal may have leaked to other parts of the image producing artifacts. Therefore, to verify that our recorded dendritic signals did come from the dendritic branches, we plotted the fluorescence image amplitude versus the change in fluorescence induced by the ‘OFF’ visual stimulus (Fig 2B–2D). For large proximal dendritic branches the change in OGB-1 fluorescence during visual stimulation was sufficiently large to have reliable values for each pixel of the image (pixel size was approximately 0.8 μm). Fig 2C shows a profile of a proximal dendrite recorded during visual stimulation inducing action potentials in the somatic recording (not shown) while in Fig 2D the absolute value of the dendritic branch image pixel fluorescence is plotted against the change in fluorescence induced by the visual OFF stimulus. There was very strong correlation between the dendritic image signal amplitude the amplitude of the fluorescence change (r^2^ = 0.80, N = 231, p <0.0001), demonstrating that most if not all fluorescence change is attributable to the calcium transients occurring inside the dendritic branch. Since such a result was obtained even for proximal dendrites, for which the risk for contamination from the somatic signal is the highest, we assume that in our dendritic recordings we did not have significant contamination from the somatic signal. In addition, as shown in Fig 1, no change in fluorescence signal during visual stimulation could be detected for background fluorescence images (light grey squares traces in Fig 1B and 1C correspondingly).

**Fig 3:**
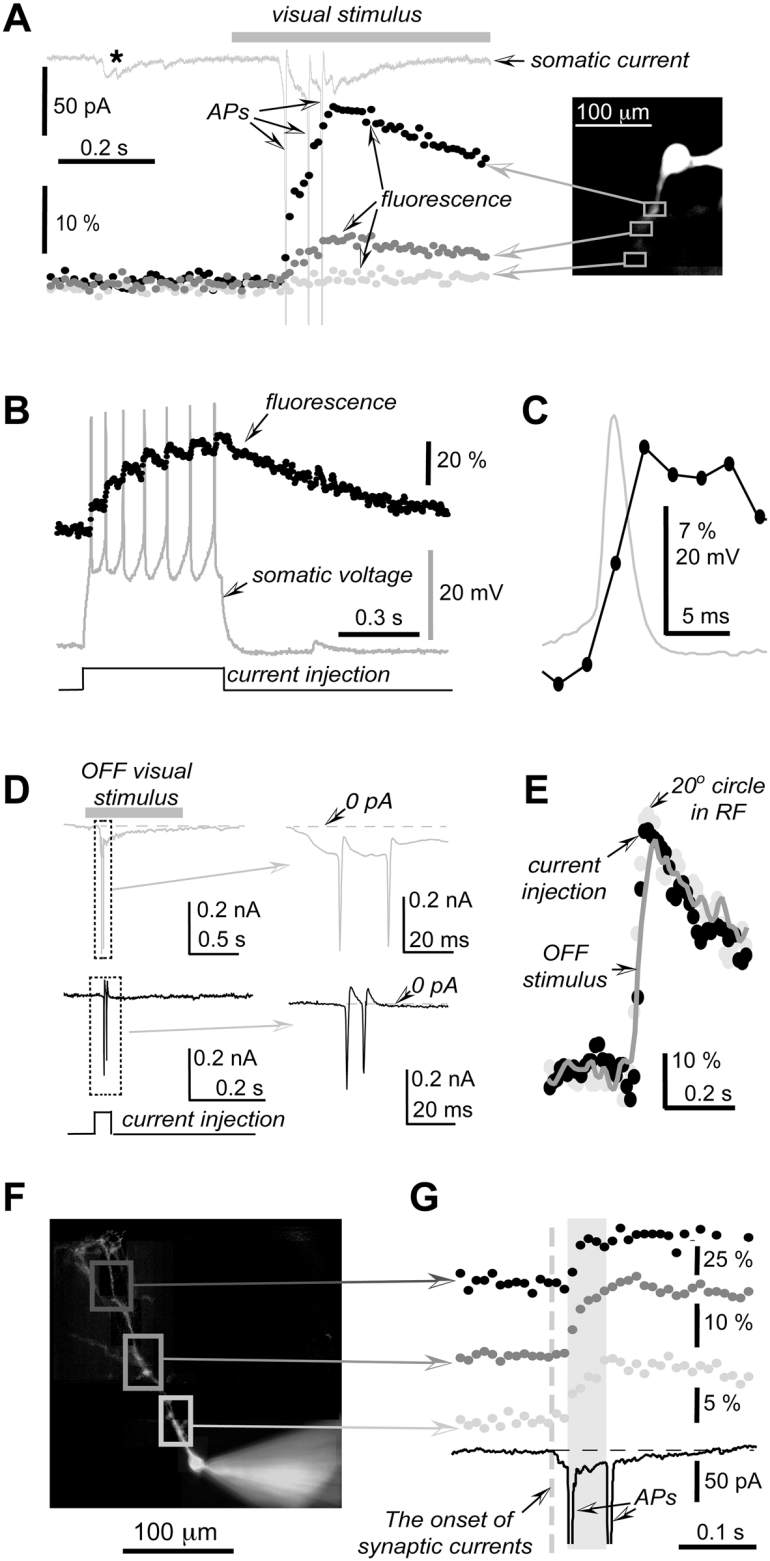
Action potentials actively propagating along dendrites are responsible for calcium concentration transients detected by OGB-1 dye. ***A***. All detected Ca_i_^2+^ increases coincided with action potentials. In this trace three action potentials correspond to three steps of OGB-1 signal increases. ***B***. During current injection all 7 action potentials caused OGB-1 fluorescence intensity increases. ***C***. The first action potential from B is shown on faster time scale with a corresponding OGB-1 fluorescence signal. It is clear that all increase in OGB-1 fluorescence occurred within few milliseconds of the action potential occurrence. The fluorescence sampling rate was 440 Hz. The vertical bar serves as a scale for both the voltage (20 mV) relative fluorescence change, dF/F, 5%. ***D E***. Calcium influx was similar during action potentials evoked by a voltage step a visual stimulus. In ***D*** current traces evoked by a visual stimulus (a ~20° wide black circle in the center of the receptive field) during voltage steps (a 40 mV, 20 ms long voltage step, bottom black traces) are shown in slow in fast time scales (left right panels correspondingly). Leak currents capacitance charging transients were subtracted for clarity. In ***E*** fluorescence traces for both conditions are aligned at the onset of the responses. Grey filled circles correspond to visually evoked stimulus while black filled circles correspond to voltage step comm. In addition, a fluorescence trace corresponding to OFF visual stimulus is shown as a dark grey line. Although no membrane currents are shown for this stimulation, two action potentials were also evoked during this OFF stimulus albeit with larger interval that explains slightly slower rate of rise of the fluorescence signal. For all three conditions the fluorescence measurements were taken in the same dendrite section at about 100 μm from the soma. ***F G***. OGB-1 fluorescence increase could be detected up to >300 μm from the soma in the area of fine dendritic branches near the edge of the optic tectum. In all three locations the onset of the OGB-1 signal increase was nearly simultaneous, probably limited by the fluorescence signal sampling rate of 100 Hz. A vertical thick grey broken line in ***G*** denotes the onset of the inward synaptic currents while the horizontal thin black broken line denotes the 0 pA baseline for the current trace.

Next, we investigated what caused these transients. We found that all large dendritic calcium concentration transients were associated with the action potentials recorded in the soma. Although we often employed voltage clamp to visualize synaptic currents, large dendritic trees remained unclamped neuronal responses to visual stimulation were frequently accompanied by action potentials on top of the synaptic currents [29, 36]. Fig 3 shows clearly that there was a perfect correlation between the occurrence of action potentials rapid changes in fluorescence, corresponding to rapid increase in calcium concentration. Fig 3A shows a fluorescence trace obtained in a response to an OFF visual stimulus a light grey trace on the top represents a somatic current recorded in the voltage-clamp mode. Fast downward deflections represent unclamped action potentials. Three lower traces shown as filled circles represent the time-course of fluorescence in three locations, which are shown in the inset image. In two locations on the dendritic tree a clear transient of fluorescence coincides well with the action potentials in the top current trace while no fluorescence signal transients can be detected in the bottom fluorescence trace taken from aside of the dendritic tree. It should be noted that a spontaneous synaptic event without action potentials indicated by an asterisk was not accompanied by a detectable fluorescence signal transient in any tested location. In Fig 3B a sample trace in current clamp with a train of action potentials evoked by a current step shows a perfect coincidence of a series of increases in fluorescence measured in a proximal dendritic tree branch at high scanning rates of 440 Hz (~2 ms per point). By plotting these traces at faster time scale for the first action potential (Fig 3C) one can see that all increase in the fluorescence intensity occurred within few milliseconds from the action potential indicating that most if not all calcium entry occurred during this single action potential. The notion that action potentials but not synaptic currents were responsible for most calcium entry into the dendrites is confirmed by experiments, in which in the same neuron the same number of action potentials were evoked either by a visual stimulus or by voltage step comm (Fig 3D and 3E). The corresponding dendritic calcium concentration transients were of the same amplitude in spite of the presence of large synaptic currents in the visual stimulus evoked response recorded in the soma (Fig 3D, top traces, compare with traces below that were obtained with voltage comm stimulation). No difference in calcium transient amplitude was found for two types of visual stimuli used here, the dimming OFF stimulus a ~20° wide black circle presented in the center of the receptive field (Fig 3E, a dark grey line light grey circles correspondingly). To match the number of action potentials evoked during each visual stimulation, the change in contrast during OFF stimulation was gradually reduced until only two action potentials were evoked, that was the same number of action potentials obtained with ~20° black circle in the center of the receptive field stimulation. These data demonstrate that no significant additional calcium ion entrance occurred due to the synaptic currents. Similar results were obtained in other two neurons. In addition, the onset of fluorescence intensity change coincided perfectly with the start of action potentials but not the onset of synaptic currents evoked by OFF stimulus along all dendritic tree length (Fig 3F and 3G), further supporting the hypothesis that all large Ca^2+^ concentration transients were caused by action potentials propagating along the dendrites.

The speed of travel of calcium signals along dendritic branches can provide further evidence on the nature of these transients because diffusional processes are very slow for distances of >100 μm [37] a small depolarization of the dendritic tree would be unable to spread widely quickly without a significant attenuation [35]. Therefore we compared the onset time in proximal distal dendritic branches. Fig 3D and 3E show that there was almost no detectable delay between the proximal the distal sections of the dendrite tested at 100 Hz of fluorescence scanning rates. We were unable to detect distal dendritic signals at higher fluorescence scanning rates because a more intense illumination was necessary for higher scanning rates, but high illumination intensity caused artifacts (see METHODS for more information). Nevertheless, these data show that the fluorescence transient signal spread over distances of >350 μm in less than 10 ms, corresponding to travel speeds of >0.03 m/s. Similar result of the virtual absence of any delay in the onset times was obtained when the proximal sections of the dendrites were tested at higher frame rates of 250–440 Hz: in all tested cases (n = 4) the onset times differed by ≤1 point of the fluorescence sampling rate regardless of the distance between the test points (up to 120 μm for 280 Hz sampling rate, corresponding to travel speeds of >0.03 m/s). The most parsimonious explanation of these results is that our fluorescence sampling rate was too slow to detect significant differences in the onset times in different locations along the dendritic branches the obtained signal propagation speed of >0.03 m/s is likely to be a gross underestimate of the real velocity, at which the observed calcium transients propagate.

**Fig 4:**
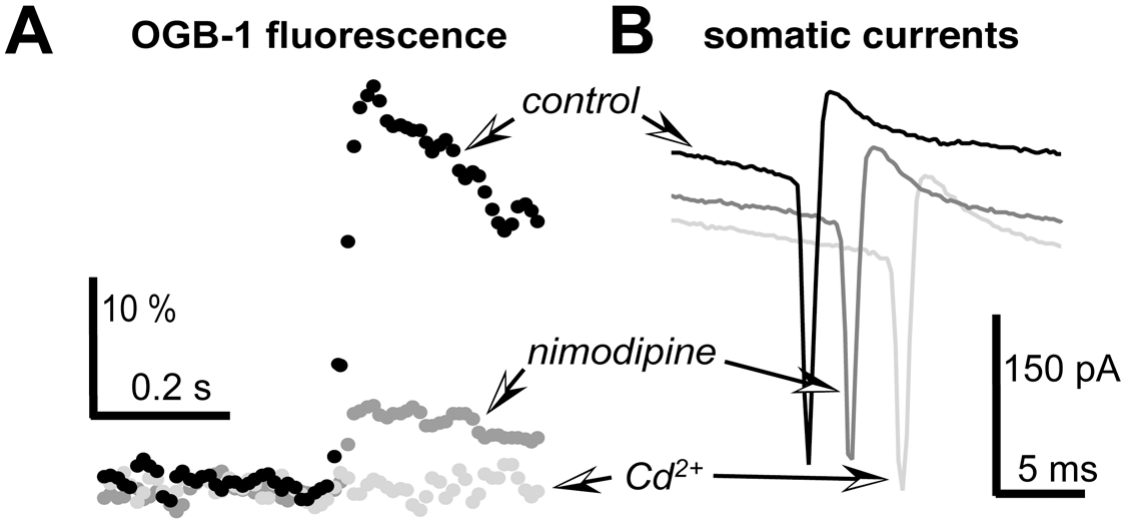
Most dendritic calcium concentration increase could be blocked by L-type calcium channel blocker nimodipine (100 μM) while the remaining small increase was abolished by application of a general calcium channel blocker Cd (0.2 μM). In this case, action potentials were evoked by voltage steps but not visual stimulus. Action potentials were shifted in time axis for better visualization while fluorescence transients were aligned to facilitate comparison.

Since sodium channels do not pass significant amounts of calcium ions under normal physiological conditions [38], the most plausible explanation of our data is that voltage-dependent calcium currents are activated during membrane depolarization caused by sodium currents when action potentials occur. To test this hypothesis, we performed pharmacological experiments. It is known that L-type calcium channel blockers do affect synaptic responses [27]. Therefore, to avoid confounding effects of synaptic transmission block, in all these pharmacological experiments, voltage steps were used to evoke action potentials. To minimize bleaching effects, we normalized each fluorescence response to the integral of the dendritic branch cross-section as shown in Fig 2C. To ensure that the reduction in the response amplitude was not caused by cell death, we made several control tests for 10–15 min to verify the stability of the response amplitude within ± 15%. As shown in Fig 4A, application of L-type calcium channel blocker significantly reduced OGB-1 fluorescence transients (to 21% of control) while current amplitude remained largely unchanged (89% of control). On average, in 5 cells the application of nimodipine (100 μM) reduced the amplitude of fluorescence transients by 74.4 ± 1.7%. Further application of Cd^2+^ (200 μM) completely obliterated fluorescence responses in two cells (0.2% of control, the action potential current amplitude remained 85% of control) suggesting that all calcium entry into the dendrites was due to activation of voltage dependent calcium channels.

## Discussion and Conclusions

We demonstrate that in the dendrites of the adult frog optic tectum visual stimuli can induce fast calcium concentration transients that propagate rapidly to distal ends of the dendrites. These large transients were always associated with the action potentials a Ca^2+^ concentration increase occurred almost entirely during a single action potential. These calcium concentration transients were reduced by >70% by L-type calcium channel blocker nimodipine eliminated entirely by a non-selective calcium channel blocker Cd^2+^. Our data is consistent with a hypothesis that the dendrites of the frog layer 6 tectal neurons can support active action potential propagation up to distal branches of the dendritic tree.

Several lines of evidence support this hypothesis. First, there was 100% correlation between the presence of action potentials in the somatic recordings large fluorescence signal transients of a calcium sensitive dye OGB-1 in the dendrites of tectal neurons. Although in some case we could detect small local dendritic OGB-1 fluorescence transients in the absence of somatic action potentials (unpublished observations, GS), for all ≥10% fluorescence changes, the onset of the calcium signal transients coincided with the action potential but not the synaptic current onset as shown in Fig 3G. This observation was very consistent for all recorded tectal neuron (n = 22). We also show that these transients were extremely rapid the whole rise in calcium concentration occurred in less than 5 ms (Fig 3C). All these facts prove that in adult frog tectal neurons all large rapid dendritic calcium concentration increases are caused by action potentials.

The large dendritic calcium signal transients were always rapidly spreading over long distances. In fact, as shown in Figs 1, 3F and 3G, these transients could be almost immediately detected over the whole image of the dendritic tree of the recorded neuron. We could only estimate the lower boundary of the calcium transient propagation velocity, which was found to be >0.03 m/s. In order to avoid the frog retina damage, for the OGB-1 dye excitation we were unable to use high illumination intensity necessary for high scanning rates no detectable changes in the fluorescence signal amplitude could be found for distal dendrites at sampling rates above 250–440 Hz. Even though the lower velocity boundary of the calcium signal propagation is likely to be a gross underestimate, a purely diffusional spread of ions is much slower cannot explain our results [37]. It would take several hundreds of milliseconds to spread calcium ions by diffusion from soma to the distal, >100 μm away from the soma, dendritic branches of the tectal neurons. It is well known that calcium buffering by fluorescent calcium ion indicators affects temporal dynamics spatial extent of the fluorescence signal [39, 40], this is especially true for high affinity indicators such as the one used in this study OGB-1, which has the estimated Kd ~200 nM [41]. Such buffering increases the decay time constants reduces the amplitude of the signal increases limits in space calcium ion diffusion. However, we observed rapid increases in the fluorescence signal, which are much less sensitive to buffering [39] the observed rapid propagation of the calcium signal transients cannot be attributed to diffusion, as it has been explained above. Therefore, our use of a high affinity calcium indicator is unlikely to have any significant impact on our main reported phenomenon here, the rapid travel along the dendrites of calcium signal transients.

Although the estimated lower boundary of the calcium signal propagation velocity, >0.03 m/s, is much lower than the reported travel speed of ~0.5 m/s of action potentials back-propagating along dendrites in the mammalian central neurons [42–44], our data suggest that active but not passive propagation was responsible for the spread of calcium signals. There is not much difference in the propagation velocity of large small action potentials [42, 44] but passive spread of depolarization is attenuated by cable properties of the dendrites [35]. It is thought that the average space constant of tectal dendrites is ~300 μm [27], suggesting that a somatic depolarization will be attenuated several fold in distal ends of the dendrites fewer calcium channels will be activated. In addition, depolarization onset will be several fold slower [35]. However, our data show that the onset is, probably, even faster in distal than proximal dendrites (Fig 3G) relative signal amplitude is unchanged throughout the whole dendritic tree.

Our reported data are somehow different from the results presented on tadpole dendritic calcium transients [17]. This study showed that both synaptic NMDA currents voltage gated calcium channels contributed to the calcium concentration transients in the distal dendritic tree branches of the tadpole tectal neurons during visual stimulation. Although we did not test NMDA receptor antagonists, a perfect correlation with the action potential but not the synaptic currents should be sufficient to rule out the synaptic current contribution to the dendritic calcium transients. We also show that both voltage-step evoked action potentials, when no synaptic currents could be detected, visually evoked responses with strong synaptic activity induced the same increase in calcium concentration that was dependent only on the number of elicited action potentials (Fig 3D and 3E). Nevertheless, it is plausible that with larger synaptic currents /or at more depolarized membrane potentials NMDA receptors could contribute to the dendritic calcium transients in the adult frog tectal neurons. Future experiments with NMDA receptor blockers stimulation protocols tuned for the NMDA receptor activation are needed to determine convincingly whether NMDA receptors can mediate calcium transients in the dendrites of adult frog tectal neurons.

In addition our data indicate much smaller dendritic calcium changes than reported by Bollman Engert (2009). In our case, on average a single action potential increased fluorescence by 40% while in the above study by >100%. At least several experimental details may account for differences in these results. First, we recorded from adult frogs with much larger dendritic trees than in tadpoles. It is likely that both the synaptic receptor properties the density the calcium buffering properties are vastly different between neurons in tadpoles adult frogs. Second, we employed whole cell recordings for all our experiments a partial wash-out could affect our results. Nevertheless, we did not notice a dramatic change in the relative amplitude of recorded calcium transients during the first 20–30 min of our recordings. Finally, most of our quantitative data relate to the proximal middle sections of the dendritic tree, such as shown in Fig 2B. Meanwhile, for the fine ends of the dendrites, such as shown by the darkest square in Fig 3F, we could detect an increase in the calcium concentration only when an integral over an area of >20x20 μm^2^ was taken even though a faint image of the dendrites was clearly visible it allowed the reconstruction of the whole dendritic tree shown in Fig 1B. Background fluorescence noise, characteristic for stard fluorescence microscopy used in this study, is likely to limit our ability to detect faint signals. In contrast, the above-mentioned study used a two-photon microscopy to determine calcium increase in separate fine branches of the dendritic tree; it is likely that calcium concentration increase is much more dramatic in these fine branches than in the middle sections of the dendritic tree.

Although several studies indicate that in the optic tectum of adult frogs action potentials might rapidly propagate along dendrites large distances [17, 45], no conclusive evidence has been obtained so far. Our data show that in tectal neurons of the adult frog most calcium signals evoked by visual stimulus are associated with action potentials are mediated by voltage-gated calcium channels. Such an ability of tectal neurons to synchronously elevate calcium concentration in most dendritic branches may dramatically increase the speed of synaptic input integration enable rapid complex computations in a relatively small brain area.
